# Intravascular Lithotripsy in the Aorta and Iliac Vessels: A Literature Review of the Past Decade

**DOI:** 10.3390/jcm14155493

**Published:** 2025-08-04

**Authors:** Nicola Troisi, Giulia Bertagna, Sofia Pierozzi, Valerio Artini, Raffaella Berchiolli

**Affiliations:** Vascular Surgery Unit, Department of Translational Research and New Technologies in Medicine and Surgery, University of Pisa, Cisanello Hospital, Via Roma 67, 56126 Pisa, Italy

**Keywords:** intravascular lithotripsy, peripheral arterial disease, early outcomes

## Abstract

**Background/Objectives**: Nowadays, intravascular lithotripsy (IVL) has emerged as a novel technique for treatment of vascular calcifications, first in coronary and then in peripheral arteries. In the current literature there is little evidence that describes IVL as an effective and safe solution in treating severe aortic and aorto-iliac calcifications. The aim of this study is to report current available data about the use of IVL in treating aortic and aorto-iliac calcified lesions and its application in facilitating other endovascular procedures. **Methods**: the present review was conducted and reported in accordance with the Preferred Reporting Items for Systematic Review and Meta-Analyses (PRISMA) Guidelines. Preliminary searches were conducted on MEDLINE and Pubmed from January 2015 to February 2025. Studies were divided into 3 main categories depending on the location of calcifications and the type of treatment: IVL in visceral and infrarenal obstructive disease (group 1), IVL in aorto-iliac obstructive disease (group 2), IVL used to facilitate other endovascular procedures. Main primary outcomes in the perioperative period were technical and clinical successes and perioperative complications. Primary outcomes at 30 days and mid-term (2 years) were overall survival, limb salvage rate, primary patency, primary assisted patency, secondary patency, and residual stenosis. **Results**: Sixteen studies were identified for a total of 1674 patients. Technical and clinical successes were 100%, with low rates of perioperative complications. Dissection rate reaches up to 16.1% in some studies, without any differences compared to plain old balloon angioplasty (POBA) alone (22.8%; *p* = 0.47). At 30 days, limb salvage and survival rates were 100%. At 2 years, primary patency, assisted primary patency, and secondary patency were 95%, 98%, and 100%, respectively, with no difference compared to IVL + stenting. **Conclusions**: IVL has emerged as a novel approach to treat severe calcified lesions in visceral and aorto-iliac atherosclerotic disease and to facilitate other endovascular procedures. This technique seems to offer satisfactory early and mid-term outcomes in terms of primary, primary assisted patency, and secondary patency with low complication rates.

## 1. Introduction

In recent years, intravascular lithotripsy (IVL) has emerged as a novel technique to treat vascular calcifications in peripheral arteries [1,2,3].

The Shockwave IVL catheter allows modifications of atherosclerotic plaque by generating calcium fractures that facilitate stent expansion and luminal gain [4,5]. For severely calcified lesions, IVL offers more advantages compared to other endovascular technologies, which are often associated with perioperative complications, such as rupture, dissections, and occlusions. Indeed, vessel compliance in diffused calcified arteries is very poor and this rigidity represents a major issue for the access of large-bore devices [6,7,8].

The efficacy and safety of IVL has already been demonstrated in coronary disease, where this device was launched on the market. Although promising in other districts, such as juxtarenal and aorto-iliac ones, this technique has been evaluated only in small and non-randomized cohorts. In addition, outcomes reported in the current literature are based on a short-term follow up, so that assessing the durability of the procedure remains challenging [9,10,11,12,13,14,15,16,17].

Therefore, the aim of this study is to report, describe and compare current available data about the use of IVL in treating aortic and aorto-iliac calcified lesions, and its potential application in facilitating other endovascular procedures.

## 2. Materials and Methods

The present review was conducted and reported in accordance with the Preferred Reporting Items for Systematic Review and Meta-Analyses (PRISMA) Guidelines [18].

The literature search strategy was carried out by the review author team (N.T., G.B., P.S., V.A.). Preliminary searches were conducted on Medical Literature Analysis and Retrieval System Online (MEDLINE) and Pubmed from January 2015 to February 2025. The Society of Vascular Surgery (SVS) and European Society for Vascular Surgery (ESVS) guidelines were also reviewed [19]. A combination of controlled vocabulary and free text terms was used to investigate the databases.

In particular, the following search strategies were used on each database:

“Intravascular lithotripsy AND vascular surgery AND short-term results” (textword).

“Intravascular lithotripsy AND vascular surgery AND mid-term results” (textword).

“Intravascular lithotripsy AND aorto-iliac AND short-term results” (textword).

“Intravascular lithotripsy AND aorto-iliac AND mid-term results” (textword).

“Intravascular lithotripsy AND aortic AND short-term results” (textword).

“Intravascular lithotripsy AND aortic AND mid-term results” (textword).

Narrative reviews and articles with few or incomplete data were excluded. Updated studies were excluded as well. Inclusion criteria involved the application of IVL in aorta or aorto-iliac district, specifying the localization and the underlying disease of the treated patients.

We divided the articles included into three main classes depending on the location of the calcifications and the aim of the treatment: IVL in visceral and infrarenal obstructive disease (group 1), IVL in aorto-iliac obstructive disease (group 2), and IVL used to facilitate other endovascular procedures (group 3).

When possible, abstracts were reviewed online and suitable articles downloaded entirely for data extraction. If abstracts were not available, a full copy of the article was assessed.

Reason for exclusion:

Reason 1: narrative review without significant data.

Reason 2: few or incomplete data.

Reason 3: updated or duplicate studies.

A meta-analysis was not feasible due to various factors, including non-randomized studies, high risk of bias, statistical heterogeneity, and the inability to meaningfully combine studies due to differences in design, interventions, and outcomes.

**Figure 1:**
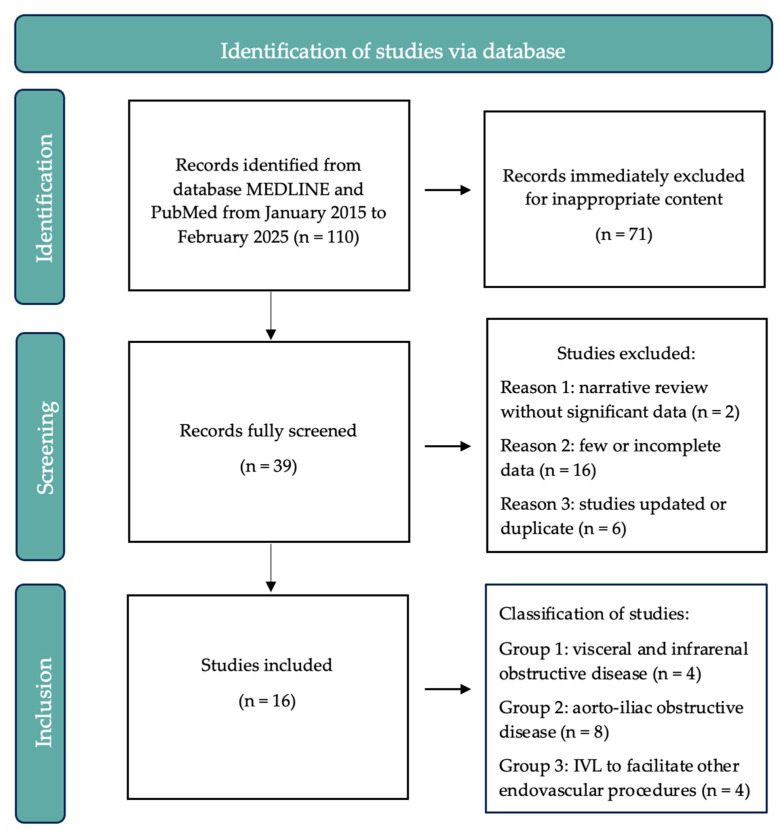
Flow-chart based on PICOS approach.

The flow-chart of the research is reported in Figure 1 based on PICOS approach.

### 2.1. Description of the Device 

The Shockwave IVL catheter (Shockwave Medical, Santa Clara, CA, USA) is a single-use, sterile, disposable catheter that contains multiple spark gap-based lithotripsy emitters along the shaft of an integrated balloon. The catheter is connected to the generator that is programmed to deliver a pre-defined number of pulses at a rate of 1 pulse/s. Following lithotripsy treatment, the IVL balloon was inflated to the vessel size using the balloon compliance chart as per the Instructions for Use [4].

The result is the modification on the plaque with the generation of calcium fractures, that facilitate stent expansion and luminal gain. For severely calcified lesions, IVL offers several advantages compared to plain balloon-based technologies and atheroablative technologies (rotational atherectomy, focal force, scoring and cutting ballons). These latter modalities of plaque modification are mainly targeted to intimal calcifications compared to medial ones. Moreover, they are often associated with perioperative complications, and data about their efficacy are still scarce and based on small sample sizes [6,7].

As opposed to these techniques, IVL seems to effectively increase arterial compliance and vessel dilation. Furthermore, using the device does not require any specific training, and the learning curve is relatively short. Additionally, the balloon opening pressure is lower, which reduces the risk of vascular injuries and distal embolization [8].

### 2.2. Definitions

Technical success was defined as the successful delivery of the IVL catheter across the target lesion, administration of IVL pulses without angiographic complications, and achieving a residual target lesion stenosis of less than 30%.

Clinical success was defined as the ability of IVL to produce a residual diameter stenosis < 50% with no evidence of in-hospital Major Adverse Cardiovascular Events (MACEs).

Primary patency was defined as freedom from clinically driven target lesion revascularization (CD-TLR) and freedom from restenosis as determined by duplex ultrasound (DUS) or angiogram ≥ 50% stenosis [4].

Primary assisted patency was defined as time to reintervention to maintain patency. Secondary patency was defined as restored flow in the treated segment after occlusion or restenosis. Amputation-free survival was defined as the time until major amputation of the index limb or all-cause death, whichever occurred first.

### 2.3. Statistical Analysis

Main primary outcomes considered for analysis in the perioperative period were technical and clinical successes and perioperative complications, such as dissection, perforation, flow interruption, distal embolization, and thrombosis. Then, primary outcomes at 30 days and mid-term (2 years) were overall survival, amputation-free survival, primary patency, primary assisted patency, secondary patency and residual stenosis. Continuous data were expressed as the mean values. Categoric data were expressed as percentages. The Pearson chi-square test was used to compare values between groups, based on the nature of the data and variables.

Statistical significance was defined at the *p* < 0.05 level.

Statistical analysis was performed using SPSS software (version 24.0 for Apple; IBM Corporation, Armonk, NY, USA).

## 3. Results

We selected a total of 110 reports. 71 were considered inappropriate for the content of the review and the remaining 39 articles were rescreened. Of those, only 16 studies fulfilled the inclusion criteria for a total of 1674 patients.

All procedure examined were performed according to the current ESVS guidelines.

Patients treated suffered from juxtarenal and infrarenal coral reef aorta, heavy-calcified aorto-iliac disease, and peripheral lesions with severe calcification before endovascular aneurysm repair (EVAR) or transcatheter aortic valve implantation (TAVI).

### 3.1. Visceral and Infrarenal Obstructive Disease

IVL in patients with juxtarenal and visceral disease seems to offer satisfactory results in both perioperative and 30-day outcomes. The rate of technical and clinical successes was 100%. No perioperative complications in terms of dissection, vessel perforation, flow limitation, distal embolization, or thrombosis occurred.

At 30 days, overall survival was 100%, as well as limb salvage and primary patency rates. Residual vessel stenosis of up to 40% was the most frequent complication.

The complete list of studies included, along with their respective outcomes, are reported in Table 1.

### 3.2. Aorto-Iliac Obstructive Disease

In aorto-iliac district IVL seems to offer similar excellent outcomes in terms of technical and clinical successes. Almost no studies reported any perioperative complications, except for one study reporting a 43% of dissection on a cohort of seven patients, and the greater Distrupt PAD III study describing around 1% of dissection, vessel perforation, and distal embolization on a total of 153 patients allocated in the IVL group [4,20].

In addition, the rate of residual stenosis after IVL reaches only 20% is almost comparable with the results obtained for the juxtarenal and visceral district.

The study by Fazzini et al. [21] reported satisfactory 2-year outcomes in terms of primary patency (*p* = 0.24), primary assisted patency (*p* = 0.43), secondary patency, amputation-free survival (*p* = 0.78), and overall survival (*p* = 0.30), with no statistically significant difference between IVL alone and IVL + stenting.

Table 2 reports in detail all included studies and their respective outcomes.

### 3.3. IVL Used to Facilitate Other Endovascular Procedures

IVL seems to be a valid tool even for improving the feasibility of other endovascular procedures, such as EVAR and TAVI. Technical success was comparable with that already reported. Clinical success was equally satisfying, except for a study on 28 patients undergoing EVAR, which described a rate of 96% [22]. Complications were uncommon, although the vessel dissection was frequent. The largest meta-analysis concerning IVL before TAVI reported a low rate of dissection, around 4% [23]. Thirty-day outcomes in terms of survival, limb salvage, and primary patency rates were as high as 100%, while residual stenosis reached up to 7%.

Table 3 summarizes all studies about IVL before EVAR and TAVI.

## 4. Discussion

IVL has emerged as an innovative approach to treat highly calcified arterial lesions. In the current literature, there is little evidence about its use in several districts, due to the heterogeneity of the atherosclerotic pathology. Therefore, collecting homogenous data and analyzing the relative outcomes remains challenging.

In any case, coral reef aorta is a rare condition, characterized by calcifications in visceral and juxtarenal aorta, which may also involve iliac axis. Conventional open surgery is still considered the traditional approach across multiple districts [26]. However, the rates of hospital stay, morbidity, reinterventions, and all-cause mortality are not negligible. Indeed, operative mortality was reported to be as high as 11.6%, and the rate of postoperative complications was reported to be up to 16% [27].

On the other hand, IVL could be an alternative and less invasive option in fragile patients, particularly when obstructions are located in visceral and juxtarenal aorta. Furthermore, IVL alone without stenting seems to be enough to restore sufficient flow in visceral aorta [9].

Lithotripsy is not only an independent treatment but seems to have a complementary role in other procedures. In fact, in case of severely calcified stenosis, IVL can help prepare vessels for either plain old balloon angioplasty (POBA) or stenting. In fact, IVL improves both stentgraft expansion and sealing, reducing the risk of infolding. In these cases, the deployment of covered stents seems to improve durability of the results gained with lithotripsy [10].

In juxtarenal obstructive disease, IVL is used in a kissing conformation so that iliac calcifications can be treated simultaneously, reducing the risk of contralateral plaque disruption and embolization. In addition, when visceral arteries are close to the calcified site, inflating balloons in visceral arteries can be useful. In this scenario, intravascular ultrasound (IVUS) can represent a mini-invasive imaging technique to visualize the characteristics of both vessel and plaque before and after revascularization [11]. Although digital subtraction angiography (DSA) remains the gold-standard imaging modality in peripheral interventions, IVUS could be an additional tool, especially in calcified vessels.

Although limited by the small sample size, our review underlines the relatively low rate of perioperative complications, and the satisfactory early and mid-term outcomes of IVL. However, residual stenosis remains an issue, and comparing outcomes between IVL and other endovascular techniques is challenging because of the heterogeneity of follow up. In fact, the articles selected evaluate outcomes from the first postoperative day to 24 months, and with different methods, such as area and diameter variations, and flow velocity. In any case, all studies reported a non-hemodynamic residual stenosis, highlighting the promising role of IVL as an additional endovascular approach.

Concerning aorto-iliac occlusive disease, IVL has been increasingly used, as it has in the femoro-popliteal segment. Aorto-iliac occlusive pathology is more commonly treated with either kissing stent or Covered Endovascular Reconstruction of Aortic Bifurcation (CERAB), which demonstrate satisfactory results. Their drawbacks include requiring large-caliber catheters and sheaths that do not easily navigate the calcified iliac axis. In this scenario, the use of lithotripsy catheters with low profile may be a valid tool.

The use of IVL for vessel preparation before stenting, or as an alternative to direct stenting, has been described in the DISRUPT PAD III Study [4]. This large study reported the superiority of IVL over POBA alone for vessel preparation. The lower inflation pressure during IVL compared to POBA seems to be one of the determining factors, leading to lower rates of dissection and a reduced need for stenting. Furthermore, a recent retrospective multicenter study, evaluating safety and efficacy of IVL treatment in calcified iliac arteries, reported the rates of primary patency and assisted primary patency at 24 months of 95% and 98%, respectively, with a secondary patency rate of 100%. Then, primary patency showed no statistically significant differences between the IVL only and IVL + stent groups [21]. These data showed promising mid-term results in terms of primary patency despite low stenting rates, thereby preserving future treatment options. Intravascular lithotripsy has also been compared with rotational and orbital atherectomy in calcified coronary lesions. A recent study reported no significant differences in the 1-year Major Adverse Cardiovascular Events (MACE) between the three techniques, concluding that there is no standard method of plaque modification, and a tailor-made approach is required [28]. A similar comparison between techniques in the aorto-iliac segment could be a field of research with the possibility to perform a randomized controlled trial (RCT).

IVL seems to also have a role in the treatment of iliac in-stent restenosis (ISR). Indeed, Troisi et al. [13] described iliac ISR related to underexpansion of a bare metal stent with wide vessel calcifications. The remodeling of calcium particles achieved with lithotripsy allowed the correct stent expansion.

As mentioned, hostile and calcified iliac arteries could create challenges in gaining adequate access for endograft delivery. IVL can facilitate these maneuvers and reduce the risk of iliac rupture and/or dissection. Indeed, Fazzini et al. [22] described a postoperative luminal gain of up to 93% at one-month CTA, along with an improvement of two Rutherford classes in all patients with aorto-iliac occlusive disease and a primary patency rate of 100% at the last follow-up. Similar satisfactory outcomes have been reported for addressing severe calcifications in hostile necks before EVAR, reducing the rate of type Ia endoleak [25]. However, these results are derived from clinical cases only and need to be verified in wider cohorts.

Furthermore, a recent meta-analysis described the safety and efficacy of IVL in preparing severe calcified peripheral arteries before TAVI. The subgroup analysis of patients who underwent TAVI with IVL assistance presented a 100% implantation success, with only 4% of dissections [23]. These data further underline the promising results of IVL not only as an independent technique, but also as a complementary technique in other procedures. For this reason, although based on low-quality evidence, intravascular lithotripsy should be integrated in current guidelines as an additional tool among atheroablative technologies. Indeed, the choice between different techniques can be at the discretion of the clinician based on availability and expertise. In any case, larger multicenter and randomized trials are still necessary to better elucidate the efficacy and durability of this technique, and to formulate clinical recommendations.

This study has some limitations that should be mentioned. First, the site and the nature of the lesions and the follow-up among studies were heterogeneous. Second, in this narrative review, patients with different comorbidities were pooled together, reducing the generalizability of results. Then, most data were extracted from clinical cases or series, reducing the power of statistical analysis. Ultimately, there were no studies comparing IVL with other techniques and none with sufficient follow-up to evaluate its long-term efficacy and safety.

## 5. Conclusions

IVL has emerged as a novel approach to treat severely calcified lesions especially in aorto-iliac diseases. This technique seems to offer satisfactory early and mid-term outcomes in terms of primary, primary assisted patency, and secondary patency, with low complication rates.

Furthermore, this paper highlights the potential role of IVL in improving the quality of proximal sealing zones, preventing type Ia endoleak, enhancing the trackability of devices and reducing major complications. With this in mind, IVL seems to be a valid tool for extending the application of standard EVAR in patients with challenging anatomies and TAVI in patients with calcified accesses. On the other hand, the effective long-term durability of IVL has yet to be investigated.

Lastly, further randomized studies with long-term follow-up and wider sample size are necessary for defining the safety and efficacy of integrating IVL in standard endovascular procedures.

## Figures and Tables

**Table 1:** Studies concerning visceral and infrarenal occlusive disease.

	Studies			
	Allevi et al. [9] J Vasc Surg Cases Innov Tech. 2024	Piazza et al. [10] J Vasc Surg Cases Innov Tech. 2023	Elger et al. [11] JACC Case Rep. 2024	Chag et al. [12] Eur Heart J Case Rep. 2021
Cohort (n)	1	2	1	1
Outcomes (%):				
Intraoperative				
- Technical success	100	100	100	100
- Clinical success	100	100	100	100
Perioperative complications				
- Dissection	0	0	0	0
- Perforation	0	0	0	0
- Flow interruption	0	0	0	0
- Distal embolization	0	0	0	0
- Thrombosis	0	0	0	0
30-day				
- Survival	100	100	100	100
- Limb salvage	100	100	100	100
- Primary patency	100	100	100	100
- Residual stenosis	20	40	0	0

**Table 2:** All included studies for aorto-iliac obstructive disease with the reported outcomes.

Study	Cohort	Outcomes	Rate %	
Troisi et al. [13] J Vasc Surg Cases Innov Tech. 2024	1	Intraoperative		
		- Technical success	100	
		- Clinical success	100	
		Perioperative complications		
		- Dissection	0	
		- Perforation	0	
		- Flow interruption	0	
		- Distal embolization	0	
		- Thrombosis	0	
		30-day outcomes		
		- Survival	100	
		- Limb salvage	100	
		- Primary patency	100	
		- Residual stenosis	20	
Honton et al. [14] JACC Cardiovasc Interv. 2020	4	Intraoperative		
		- Technical success	100	
		- Clinical success	100	
		Perioperative complications		
		- Dissection	0	
		- Perforation	0	
		- Flow interruption	0	
		- Distal embolization	0	
		- Thrombosis	0	
		30-day outcomes		
		- Survival	100	
		- Limb salvage	100	
		- Primary patency	100	
		- Residual stenosis	0	
Konstantinou et al. [15] J Endovasc Ther. 2024	1	Intraoperative		
		- Technical success	100	
		- Clinical success	100	
		Perioperative complications		
		- Dissection	0	
		- Perforation	0	
		- Flow interruption	0	
		- Distal embolization	0	
		- Thrombosis	0	
		30-day outcomes		
		- Survival	100	
		- Limb salvage	100	
		- Primary patency	100	
		- Residual stenosis	0	
Misuraca et al. [16] Cureus. 2024	1	Intraoperative		
		- Technical success	100	
		- Clinical success	100	
		Perioperative complications		
		- Dissection	0	
		- Perforation	0	
		- Flow interruption	0	
		- Distal embolization	0	
		- Thrombosis	0	
		30-day outcomes		
		- Survival	100	
		- Limb salvage	100	
		- Primary patency	100	
		- Residual stenosis	0	
Zenunaj et al. [17] Vascular. 2025	1	Intraoperative		
		- Technical success	100	
		- Clinical success	100	
		Perioperative complications		
		- Dissection	0	
		- Perforation	0	
		- Flow interruption	0	
		- Distal embolization	0	
		- Thrombosis	0	
		30-day outcomes		
		- Survival	100	
		- Limb salvage	100	
		- Primary patency	100	
		- Residual stenosis	0	
Radaideh et al. [20] Vascular disease management. 2019	7	Intraoperative		
		- Technical success	100	
		- Clinical success	100	
		Perioperative complications		
		- Dissection	43	
		- Perforation	0	
		- Flow interruption	0	
		- Distal embolization	0	
		- Thrombosis	0	
		30-day outcomes		
		- Survival	100	
		- Limb salvage	100	
		- Primary patency	100	
		- Residual stenosis	0	
Tepe et al. [4] JACC Cardiovasc Interv. 2022	306			vs. PTA (*p*)
		Intraoperative		
		- Technical success	90.1	64.5 (<0.0001)
		- Clinical success	100	100
		Perioperative complications		
		- Any dissection	16.1	22.8 (0.47)
		- Flow limiting dissection	0	0 (>0.99)
		- Perforation	0	1.3 (0.53)
		- Flow interruption	0	0
		- Distal embolization	0	0.6
		- Thrombosis	0	0
		30-day outcomes		
		- Survival	100	100
		- Limb salvage	100	100
		- Primary patency	99.4	98.1
		- Residual stenosis	21.5	20.7 (0.39)
Fazzini et al. [21] J Vasc Surg. 2025	86			IVLvs IVL + stent (*p*)
		Intraoperative		
		- Technical success	99	-
		- Clinical success	98	-
		Perioperative complications		
		- Flow limiting dissection	5	-
		- Perforation	1	-
		- Flow interruption	0	-
		- Distal embolization	0	-
		- Thrombosis	-	-
		- Access site	1.7	-
		30-day outcomes		
		- Survival	100	
		- Limb salvage	100	
		- Residual stenosis	5–15	(0.08)
		Mid-term (two-year) outcomes		
		- Primary patency	95	(0.24)
		- Assisted primary patency	98	(0.43)
		- Secondary patency	100	
		- Amputation-free survival	90	(0.68)
		- Overall survival	91	(0.30)

**Table 3:** Studies concerning the use of IVL to facilitate other endovascular procedures.

	Studies			
	Fazzini et al. [22] Endovasc Ther. 2024	Khalid et al. [24] JACC Cardiovasc Interv. 2019	Mastropaolo et al. [25] EJVES Vasc Forum. 2024	Sagris et al. [23] Vasa. 2024
Cohort (n)	28	1	1	1223
Outcomes (%):				
Intraoperative				
- Technical success	100	100	100	100
- Clinical success	96	100	100	100
Perioperative complications				
- Dissection	0	0	40	4
- Perforation	0	0	0	0
- Flow interruption	0	0	0	0
- Distal embolization	0	0	0	0
- Thrombosis	0	0	0	0
30-day				
- Survival	100	100	100	-
- Limb salvage	100	100	100	-
- Primary patency	100	100	100	-
- Residual stenosis	-	0	-	7
